# Male-killing *Wolbachia* do not protect *Drosophila bifasciata* against viral infection

**DOI:** 10.1186/1471-2180-12-S1-S8

**Published:** 2012-01-18

**Authors:** Ben Longdon, Daniel K Fabian, Gregory DD Hurst, Francis M Jiggins

**Affiliations:** 1 Institute of Evolutionary Biology, and Centre for Immunity, Infection and Evolution, University of Edinburgh, Ashworth Labs, Kings Buildings, West Mains Road, Edinburgh, EH9 3JT, UK; 2 Institute of Integrative Biology, University of Liverpool, Liverpool, L69 7ZB, UK; 3 Department of Genetics, University of Cambridge, Cambridge, CB2 3EH, UK; 4 Current address: Institute of Population Genetics, Vetmeduni Vienna, Veterinaerplatz 1, A-1210 Vienna, Austria

## Abstract

**Background:**

Insect symbionts employ multiple strategies to enhance their spread through populations, and some play a dual role as both a mutualist and a reproductive manipulator. It has recently been found that this is the case for some strains of *Wolbachia*, which both cause cytoplasmic incompatibility and protect their hosts against viruses. Here, we carry out the first test as to whether a male-killing strain of *Wolbachia* also provides a direct benefit to its host by providing antiviral protection to its host *Drosophila bifasciata*. We infected flies with two positive sense RNA viruses known to replicate in a range of *Drosophila* species (Drosophila C virus and Flock House virus) and measure the rate of death in *Wolbachia* positive and negative host lines with the same genetic background.

**Results:**

Both viruses caused considerable mortality to *D. bifasciata* flies, with Drosophila C virus killing 43% more flies than the uninfected controls and Flock House virus killing 78% more flies than the uninfected controls. However, viral induced mortality was unaffected by the presence of *Wolbachia*.

**Conclusion:**

In the first male-killing *Wolbachia* strain tested for antiviral effects, we found no evidence that it conferred protection against two RNA viruses. We show that although antiviral resistance is widespread across the *Wolbachia* phylogeny, the trait seems to have been lost or gained along some lineages. We discuss the potential mechanisms of this, and can seemingly discount protection against these viruses as a reason why this symbiont has spread through *Drosophila* populations.

## Background

Maternally transmitted bacterial symbionts are extremely common in insects, with over half of all species estimated to be infected by bacteria from the genus *Wolbachia* alone [1]. Because maternal inheritance is often imperfect, and there is commonly a direct physiological cost to infection associated with presence of the bacteria, these infections can only be maintained where they increase either the survival or production of female hosts [2]. Some symbionts become parasites that manipulate the reproduction of their hosts to enhance their own transmission [3]. For example, many distort the sex ratio of the host towards females — the transmitting sex — to aid their spread [4-6]. Others act as mutualists, increasing the survival or reproductive success of their hosts, and therefore the number of offspring to which they are transmitted [7]. Some mutualists are essential for the host to survive and reproduce (primary symbionts) [8], while others play non-essential facultative roles and typically only infect a subset of the population (secondary symbionts [7,9]).

A number of recent studies have found secondary symbionts providing the host with protection against parasites and pathogens [10]. In aphids various bacterial symbionts confer protection to parasitoid wasps [11-13] and fungi [14], while *Spiroplasma* bacteria provide protection from nematodes in *Drosophila neotestacea *[15] and parasitoids in *Drosophila hydei *[16]*.* Recently, *Wolbachia* has been shown to make species of *Drosophila* and mosquitoes resistant to RNA viruses [17-22]. It can also make *D. melanogaster* more tolerant to viral infection, as the survival of flies infected with flock house virus (FHV) increased despite there being no effect on viral titres [18]. This protection against viruses is effective against a remarkably diverse range of single-stranded positive-sense RNA viruses, including; *Dicistroviridae* (*Drosophila C virus* and *Cricket paralysis virus*), *Nodaviridae* (*Flock House virus*), Picorna-like viruses (Nora virus), *Togaviridae* (*Chikungunya virus*) and *Flaviviridae* (*Dengue virus* and *West Nile virus*) [17,18,20,22,23].

Symbionts can sometimes employ multiple strategies to enhance their spread through populations. *Rickettsia* in whiteflies act both to directly increase host fitness and distort the sex ratio towards the production of female offspring [24]. It has recently been shown that the same strain of *Wolbachia* can both act as both a mutualist and a reproductive manipulator; in *Drosophila simulans*, strains of *Wolbachia* that induce strong cytoplasmic incompatibility also protect the host from viral infection [19]. Such dual strategies have the potential to explain several puzzling aspects of symbiont biology. For example, symbionts that cause cytoplasmic incompatibility are extremely common, despite them only being able to invade populations when they exceed a threshold prevalence [2,25,26]. This restrictive condition for invasion can disappear if the bacterium is also a mutualist [2]. If symbionts are maintained in populations by cytoplasmic incompatibility, theory predicts that there are no stable equilibria below 50%, and yet observed prevalence for *Wolbachia* in *D. melanogaster* are commonly below 50% [27,28]. This has led to the prediction that such symbionts must also carry some unknown benefit to host fitness [29], and recent models have suggested natural enemy resistance can both eliminate any threshold for invasion and stabilize low prevalence *Wolbachia* infections [30]. Similarly, male-killing bacteria spread when the death of a male benefits its sisters who will transmit the infection, and this will only occur when there are antagonistic sibling interactions such as cannibalism or competition [4]. However, some male-killers have been reported from species where eggs are laid singly [31], so sibling interactions are of low intensity. Again, this could be explained if these bacteria have other effects, such as increasing host resistance to pathogens. The high prevalence of symbionts within and across species [32] could therefore be result of such symbionts that ‘employ’ multiple strategies, and may help explain their apparent success in invading new host populations or host species.

In this study we have tested whether *D. bifasciata* infected with a male-killing strain of *Wolbachia* have greater protection from viral pathogens. This strain of *Wolbachia* naturally infects 5-7% of female *D. bifasciata* resulting in close to 100% female broods at 18°C [33]. At elevated temperatures, infected males can be produced, and then the bacteria cause weak cytoplasmic incompatibility when crossed to uninfected females [33]. In this study we examine whether this bacterium has a third phenotype by testing whether it confers protection from two RNA viruses.

The first virus we used was Drosophila C virus (DCV), a positive sense RNA virus in the family *Dicistroviridae *[34] that naturally infects *D. melanogaster* in the wild [35,36]. DCV commonly infects laboratory stocks of other *Drosophila* species [37], and can replicate when injected into a wide range of insects [38]. Secondly we used *Flock House virus* (FHV), a positive sense RNA virus in the family *Nodaviridae *[39]. It is not a natural pathogen of *Drosophila* (having been isolates from a coleopteran [40]), but will replicate in a broad range of insects and other taxa [41-44]. *Wolbachia* has been reported to increase the survival of *D. melanogaster* infected with both of these viruses [17,18].

## Methods

The *Wolbachia*-infected line of *Drosophila bifasciata* was collected in Japan in 1998 [33]. Since then (>140 generations) they have since been maintained by backcrossing infected females to males from an isofemale uninfected line present in the lab for 20 years. The two lines therefore have the same nuclear genetic background. Because infected flies were maintained using male flies from the uninfected stock, other aspects of the flies (such as any commensal flora) will also be similar. The *Wolbachia* infection rate was 100% (no males were observed in the infected line). The flies were reared on agar-malt medium at ~18°C.

We used reverse transcription (rt) PCR to check that the fly stocks we were using were not infected with DCV or FHV before the experiment. Total RNA was extracted from 40 individuals per line using Trizol reagent (Invitrogen Corp, San Diego, CA, USA) as described previously [45]. RNA was then reverse-transcribed with Promega Goscript reverse transcriptase (Promega Corp, Madison, WI, USA) using random hexamer primers. PCR was carried out on each line using DCV (kindly provided by Darren Obbard) and FHV primers [46] (DCV1290F 5’- GATGGTGTTGGCTCTGAACAGATG-3’, DCV1590R 5’-CAACTGTATCTTCCAATGCACCCTG-3’ FHV RNA 1&3 F 5'-GGACCGAAGTGCGGTGATG-3', FHV RNA 1&3 R 5′-CAGTTTTGCGGGTGGGGGG-3′) with a touchdown PCR cycle and the viral isolates used for injections as positive controls. The FHV primer pair are located in conserved regions (based on alignment to the related Black Beetle virus and Boolara virus) as are the DCV primers (based on an alignment to another DCV isolate: Darren Obbard personal communication) so should amplify any similar viruses if present.

We then tested the effect of fly Wolbachia infection status on viral pathogenicity. The viral isolates have been described previously [36,46] (kindly provided by Luis Texiera) and were prepared as in [18]. We injected virgin females aged between 4 and 10 days old with 69nl of virus into the abdomen of the fly using a Nanoject II (Drummond scientific, Bromall, PA, USA). The viruses were injected at a tissue culture infective dosage_50_ of 1.35 x 10^6^ TCID_50_ in 69nl for FHV and 1000 TCID_50_ in 69nl for DCV.

To produce the virus, Schneider Drosophila line 2 (DL2) cells were cultured at 26.5°C in Schneider’s Drosophila Medium (Invitrogen) supplemented with 10% Fetal Bovine Serum, 2mM L-Glutamine, 100 U/ml penicillin, and 100 μg/ml streptomycin (all Invitrogen). The cells were infected with DCV, and after they showed cytopathic effect they were filtered through a 0.45 μm filter and centrifuged at 13.500 rpm for 10 minutes to remove any bacteria or cellular components. Aliquots of a 10^-4^ dilution of the virus suspension were prepared using 50 mM TE buffer and frozen at -80°C. To calculate the infectivity of the virus, the Tissue Culture Infective Dose 50 (TCID_50_) was calculated. Starting from the 10^-4^ dilution, serial dilutions to 10^-10^ were made in Schneider’s medium, and each dilution was added to 8 wells of a plate. After 7 days the wells were examined and classed as “infected” when cell death and cytopathic effects were clearly visible. The TCID_50_ was calculated by the Reed-Muench end-point method [47]. The Poisson distribution was used to get the number of infective units per ml (IU/ml) [48]. The experiment was done twice to ensure the estimates of the TCID_50_ were consistent.

As a negative control we also injected flies with Drosophila Ringer’s solution [49] for the DCV experiment and Drosophila Ringer’s solution diluted 1:2 with Tris 50mM pH 7.5 for the FHV experiment. The different negative controls reflect how the viral isolate was diluted. After injection, flies were kept in vials of agar-sugar medium at ~18°C.

The flies were examined each day and the number of dead individuals in each vial was recorded. The effect of *Wolbachia* on survival rates was analysed using a Cox’s proportional hazards mixed effect model, which accounted for between vial variation in survival rates. The hazard for the *i*th individual from vial *j* at time *t* was modelled as:

Where *H*_0_(*t*) is the baseline hazard at time *t*, *X_i_* is a vector of the fixed effects, β is the corresponding vector of coefficients, and *b_j_* is a random effect of vial *j.* The fixed effects consisted of treatment (virus or negative), *Wolbachia* (infected or uninfected) and their interaction. Flies alive at the end of the experiment were censored. The model was fitted by maximum likelihood using the coxme package in R (R Foundation for Statistical Computing, Vienna, Austria).

## Results and discussion

**Figure 1:**
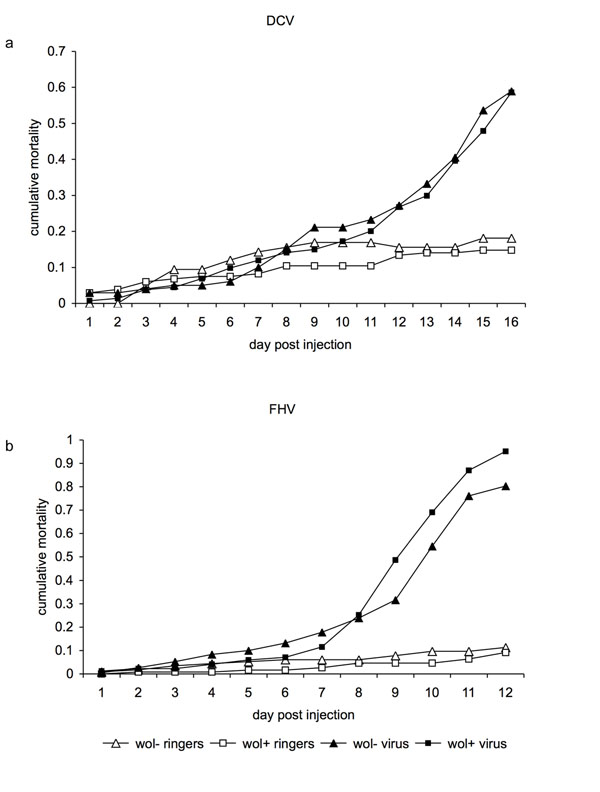
Cumulative mortality following injection with DCV (a) or FHV (b). Flies were *Wolbachia* infected (squares) or uninfected (triangles). Filled points represent viral injected and unfilled points control injected flies.

DCV: Having established that neither of the *D. bifasciata* lines tested positive for DCV-like viruses by rtPCR, 454 flies were injected with DCV (Additional file 1), and their mortality recorded over 16 days (Figure 1a). DCV caused considerable mortality (*z*=-4.32, *P*<0.001), with the death rate of infected flies accelerating after ten days, such that 59% of the DCV injected flies had died by day 16 in comparison to 16% in the uninfected controls. However, the presence of *Wolbachia* did not affect the rate at which DCV kills flies (*Wolbachia* x treatment interaction: *z*=0.23, *P*=0.82), nor was there an overall effect of *Wolbachia* on survival (*z*=0.51, *P*=0.61).

FHV: The results from the FHV experiment were similar. In this experiment 539 flies were injected (Additional file 1), and their mortality recorded over 12 days (Figure 1b). At the end of this time period 88% of the FHV infected flies were dead compared to 10% of the uninfected controls (*z*=-8.72, *P*<0.001). Again the presence of *Wolbachia* had no affect on the rate at which FHV killed flies (*Wolbachia* x treatment interaction: *z*=0.95, *P*=0.34), nor was there any main effect of *Wolbachia* (*z*=-0.29, *P*=0.77). Neither of the fly lines tested positive for FHV-like viruses by rtPCR.

It has recently become clear that secondary symbionts have often evolved multiple strategies to spread through host populations, and tests on a small number of *Wolbachia* strains have suggested that they may commonly play a dual role as a mutualist and reproductive parasite [19]. For the first time we have tested a male-killing strain of *Wolbachia* for antiviral effects, and we found it does not protect its host from the two RNA viruses we used. The number of other *Wolbachia* strains that have been examined for antiviral effects is still small, but the majority of these have provided protection against viruses. For example, in *Drosophila*, of the five *Wolbachia* strains tested, three have antiviral effects (*w*Mel and the mutant *w*MelPop from *D. melanogaster*, and *w*Au and *w*Ri from *D. simulans*) [17-19]. Our results suggest that *Wolbachia* strains that do not protect their hosts against viruses may be common, and that each strain will require independent evaluation.

There are a number of possible explanations as to why many *Wolbachia* strains provide antiviral protection, whereas the *D. bifasciata* male killer strain does not. The difference could be caused by genes in the host, but results from other species suggest that this may not be the most likely explanation, as *wMel* retained its antiviral effect even when it was transferred between different dipteran families [20]. We may also have picked two viruses not affected by this strain of bacterium, but again results from other *Wolbachia* strains suggest that protection is effective against a diverse range of RNA viruses with positive sense genomes [17,18,20,23]. Therefore, perhaps the most likely reason that the *D. bifasciata* male killer may lack the antiviral effect seen in other strains is due to genetic factors in the bacteria. Phylogenies of *Wolbachia* place the *D. bifasciata* male killer within the A clade, along with the other *Wolbachia* strains in *Drosophila* that offer protection against viruses [33,50,51]. In contrast, the *Wolbachia* strains from mosquitoes with antiviral effects belong to the B clade [21,23]. The lack of association between this trait and the bacterial phylogeny suggests that the trait has been lost or gained on some lineages. This is unsurprising as the *Wolbachia* genome is known to recombine [52,53] and contains mobile phage [54]. In *Hamiltonella defensa*, the only case where the genetic basis of symbiont-mediated protection is known, a protection of aphids from parasitoid wasps is encoded on genes carried by a phage [55].

Regardless of whether host or bacterial genes determine whether different strains have antiviral effects, it is possible that these genes may not encode the antiviral factors themselves, but may simply control bacterial density. In both *D. simulans *[19] and *Aedes albopictus *[22] the *Wolbachia* strains offering the greatest protection to viruses have significantly greater densities of *Wolbachia* than those that did not.

In many cases the spread of male-killing bacteria through host populations is surprising. Male-killing bacteria are only expected to invade insect populations when the death of males benefits the surviving females who will transmit the infection to their offspring [4]. For example, the females may gain resources by eating their dead brothers or avoiding competing with them for resources. In species like ladybird beetles, the eggs are laid in clutches and there are strong antagonistic interactions between siblings. In other species, like *Drosophila* and some butterflies [31], the benefits of killing males are less obvious and it is possible that the bacteria may employ other strategies to aid their spread. However, we have found that in the case of *D. bifasciata* it seems the spread of the male-killer has not been aided by any antiviral effect against the two viruses examined here.

## Author contributions

BL, GDDH and FMJ designed the study. BL and DKF carried out the experimental work. BL and FMJ analysed the data and drafted the manuscript with comments from GDDH and DKF. All authors read and approved the final manuscript.

## Competing interests

The authors declare they have no competing interests.

## Supplementary Material

Additional file 1 Number of flies injected per treatment, figure in brackets is number of vials per treatment. There was a mean of 19 flies per vial. Click here for file
