# Supplementary material for: Male-killing Wolbachia do not protect Drosophila bifasciata against viral infection
Source: BMC Microbiol. 2012 Jan 18;12(Suppl 1):S8. doi: 10.1186/1471-2180-12-S1-S8 (PMC3287519; doi:10.1186/1471-2180-12-S1-S8)
Supplement: Additional file 1 — Number of flies injected per treatment, figure in brackets is number of vials per treatment. There was a mean of 19 flies per vial. [file 1471-2180-12-S1-S8-S1.pdf]

Additional file 1

	Wolbachia uninfected control injected	Wolbachia infected control injected	Wolbachia uninfected virus injected	Wolbachia infected virus injected
DCV	84 (5)	138 (7)	99 (5)	133 (7)
FHV	107 (6)	113 (6)	155 (8)	166 (8)

Number of flies injected per treatment, figure in brackets is number of vials per treatment. There was a mean of 19 flies per vial.
